# Chagas Disease Serological Test Performance in U.S. Blood Donor Specimens

**DOI:** 10.1128/JCM.01217-19

**Published:** 2019-11-22

**Authors:** Jeffrey D. Whitman, Christina A. Bulman, Emma L. Gunderson, Amanda M. Irish, Rebecca L. Townsend, Susan L. Stramer, Judy A. Sakanari, Caryn Bern

**Affiliations:** a Department of Laboratory Medicine, University of California, San Francisco, San Francisco, California, USA; b Department of Pharmaceutical Chemistry, University of California, San Francisco, San Francisco, California, USA; c Department of Epidemiology and Biostatistics, University of California, San Francisco, San Francisco, California, USA; d Scientific Affairs, American Red Cross, Gaithersburg, Maryland, USA; Mayo Clinic

**Keywords:** Chagas disease, *Trypanosoma cruzi*, blood donors, diagnostics, serology, United States

## Abstract

Chagas disease affects an estimated 300,000 individuals in the United States. Diagnosis in the chronic phase requires positive results from two different IgG serological tests. Three enzyme-linked immunosorbent assays (ELISAs) (Hemagen, Ortho, and Wiener) and one rapid test (InBios) are FDA cleared, but comparative data in U.S. populations are sparse. We evaluated 500 seropositive and 300 seronegative blood donor plasma samples. Country of birth was known for 255 seropositive specimens, which were grouped into regions as follows: Mexico (*n* = 94), Central America (*n* = 88), and South America (*n* = 73). Specimens were tested by the four FDA-cleared IgG serological assays. Test performance was evaluated by two comparators and latent class analysis. InBios had the highest sensitivity (97.4% to 99.3%) but the lowest specificity (87.5% to 92.3%). Hemagen had the lowest sensitivity (88.0% to 92.0%) but high specificity (99.0% to 100.0%). The level of sensitivity was intermediate for Ortho (92.4% to 96.5%) and Wiener (94.0% to 97.1%); both had high specificity (98.8% to 100.0% and 96.7% to 99.3%, respectively). The levels of antibody reactivity and clinical sensitivity were lowest in donors from Mexico, intermediate in those from Central America, and highest in those from South America. Our findings provide an initial evidence base to improve laboratory diagnosis of Chagas disease in the United States. The best current testing algorithm would employ a high-sensitivity screening test followed by a high-specificity confirmatory test.

## Abstract (precis)

Chagas disease affects an estimated 300,000 individuals in the United States. Diagnosis in the chronic phase requires positive results from two different IgG serological tests. Three enzyme-linked immunosorbent assays (ELISAs) (Hemagen, Ortho, and Wiener) and one rapid test (InBios) are FDA cleared, but comparative data in U.S. populations are sparse. We evaluated 500 seropositive and 300 seronegative blood donor plasma samples.

## INTRODUCTION

Chagas disease is the most important tropical disease in the Americas. The attributable disease burden in the region, based on disability-adjusted life years lost, is nearly 8 times greater than that due to malaria and 20% higher than that for dengue (1). An estimated 6 million people, predominantly in Mexico, Central, and South America, are currently infected with Trypanosoma cruzi (2). Chronic infection persists lifelong in the absence of treatment, with tissue tropism for cardiac myocytes and the enteric nervous system (3–6). Over time, 20% to 30% of infected individuals develop cardiac or gastrointestinal disease. Widespread enzootic transmission cycles involving wildlife and sylvatic triatomine vectors occur in the United States, but autochthonous T. cruzi transmission to humans appears to be very rare (7, 8). Locally acquired infections are greatly outnumbered by the estimated 300,000 infected immigrants from Latin America residing in the United States (9, 10). The U.S. Food and Drug Administration (FDA) approved benznidazole, the first-line Chagas disease treatment, in 2017, increasing the need for reliable diagnostic testing for both individual and public health needs in the United States (11).

In the chronic phase, confirmed diagnosis requires positive results by two serological tests for IgG antibodies to T. cruzi, preferably based on different antigens (12). Currently, four serological assays, namely, the Ortho T. cruzi enzyme-linked immunosorbent assay (ELISA) (Ortho Clinical Diagnostics, Raritan, NJ), Hemagen Chagas’ kit ELISA (Hemagen Diagnostics, Inc., Columbia, MD), Wiener Chagatest Recombinante v.3.0 ELISA (Wiener Laboratories, Rosario, Argentina), and InBios Chagas Detect Plus (CDP) rapid test (InBios International, Inc, Seattle, WA), are cleared by the FDA for diagnostic use (13). The Ortho and Hemagen ELISAs are based on native parasite proteins (14–16). The other two assays are based on recombinant proteins. The Wiener ELISA uses trypomastigote-shed acute-phase antigens (SAPA) and recombinant epimastigote antigens 1, 2, 13, 30, and 36 (17). The InBios test is based on the recombinant multiepitope fusion antigen ITC8.2 (18). All four assays report high sensitivity and specificity in their FDA 510(k) clearance applications (reported percent sensitivity/specificity: Ortho, 98.9/99.99; Hemagen, 100/98.7; Wiener, 99.3/98.7; InBios, 95 to 100/87 to 98). However, comparative performance data are lacking for at-risk populations in the United States, as well as for those in Mexico and Central America, the predominant regions of origin of U.S. immigrants (19). Emerging evidence suggests variation in test sensitivity by geographic location and a high rate of discordance between serological test results, particularly in Mexico (20–23). Comprehensive studies are needed to provide the basis for development of reliable testing algorithms. In this study, we compared the performances of the four FDA-cleared serological tests in specimens from U.S. blood donors to provide the first systematic evidence to improve laboratory diagnosis of Chagas disease in the United States.

## MATERIALS AND METHODS

### Ethical approval.

This study was approved by the American Red Cross (ARC) institutional review board and was deemed exempt from review by the Human Research Protection Program at the University of California, San Francisco (UCSF).

### Sample selection and preparation.

We evaluated archived plasma samples from 800 blood donations (BDs) collected by the ARC between September 2006 and June 2018. Specimen selection was based on confirmed T. cruzi infection status in ARC BD testing algorithms at the time of blood donation (8). ARC provided a list of 1,091 seropositive specimens, defined by repeat reactive results generated by an FDA-licensed screening test (Ortho ELISA or Abbott PRISM [Abbott Laboratories, Abbott Park, IL]) followed by confirmed-positive results generated by a supplemental test (radioimmunoprecipitation assay [RIPA], performed by Quest Diagnostics [Chantilly, VA], or Abbott enzyme strip assay [ESA]) (8). We prioritized selection of BD-positive specimens with country-of-birth data; the remainder of the 500 BD-positive specimens were selected at random. A random sample of 300 specimens was compiled from a list of 3,938 seronegative blood donations, frequency matched by region of donation to the BD-positive specimen set. No country-of-birth data were available for seronegative specimens.

Donated plasma units from each donation were frozen at –20°C within 24 h of collection. Plasma units retrieved from ARC collections used for research purposes were thawed in a temperature-controlled water bath, divided aliquots and placed into multiple tubes, and refrozen. Aliquots tested by Hemagen, Wiener, and InBios assays at UCSF were thawed and refrozen only once. For the current analysis, the Ortho ELISA was rerun on all 800 specimens in 2019. Aliquots used for current Ortho testing were thawed and refrozen twice.

Ortho ELISA testing for this study was conducted at Innovative Blood Resources, Minneapolis, MN, using the fully automated Ortho Summit system (24). The Ortho ELISA has FDA approval for blood donation screening and clearance for diagnostic purposes but is not yet marketed for the latter use. For the Ortho ELISA, signal-to-cutoff (S/CO) ratios of 1.00 or greater are considered representative of reactive results; in the blood donation screening algorithm, all reactive units are retested two more times. A blood donation is considered repeat reactive if at least 2 of 3 sample results have an S/CO ratio greater than 1.00.

Hemagen ELISA, Wiener ELISA, and InBios rapid tests were conducted at UCSF. Plasma samples were thawed at 4˚C and spun at 2,300 relative centrifugal force for 10 min to pellet any precipitate. Samples were divided into aliquots and placed at randomly assigned positions in 96-deep-well plates to blind readers performing the InBios rapid test. Plasma aliquots of 10 μl (Hemagen and Wiener) or 5 μl (InBios) were tested and interpreted in accordance with package inserts using the kit reagents, a ELx405 Select microplate washer (BioTek, Winooski, VT), and a SpectraMax Plus 384 microplate reader (Molecular Devices, San Jose, CA). The InBios package insert defines any visible test line as representative of a positive result. For quantification of the results this assay, a set of 7 quality control samples was used to construct a semiquantitative scale ranging from 0 (negative) to 6 (strongly positive) (see Fig. S1 in the supplemental material). InBios test results were scored by two independent readers blind to other assay results. The only deviation from package insert protocols was the use of plasma for Hemagen tests; the manufacturer recommends use of serum only.

### Data analysis.

We conducted three analyses to assess diagnostic test performance. Two analyses compared assay results to different reference standards: classification in prior BD testing (8) and a consensus classification based on positive results by two or more diagnostic assays in the current study. For InBios testing, reader 1 scores were used for performance calculations, and reader 2 scores were used to calculate interreader agreement statistics. The Hemagen and Wiener kits both include an indeterminate zone; results that fell in this zone were included as positive in the performance analyses, because they would necessitate confirmatory testing in real-world scenarios. This definition may overestimate the sensitivity and/or specificity of these two tests (depending on whether the gray-zone results predominantly correspond to seropositive or seronegative specimens). Exact binomial 95% confidence intervals (CI) were calculated for each of the performance parameters. Analyses were conducted in SAS 9.4 and R version 3.5.2.

The third performance assessment consisted of a latent class analysis (LCA). LCA comprises a group of mathematical modeling techniques developed to evaluate diagnostic tests in the absence of a true gold standard (25–28). We assumed two latent classes and conditional independence of test outcomes. We used bootstrapping to generate multiple samples from the data set and then applied an expectation-maximization (EM) algorithm to estimate sensitivity and specificity for each test. The distributions of the bootstrapped samples were used to generate 95% CIs. We tested the robustness of the two-class assumption by comparing fit between models assuming two versus three latent classes, using the Akaike information criterion (AIC) and Bayesian information criterion (BIC). The latent class analysis was conducted in R version 3.5.2 and RStudio version 1.1.463 using the BayesLCA package (29).

## RESULTS

California and the southeastern states accounted for nearly three-quarters of the blood donations included in the study (Table 1). BD-positive specimens were significantly more likely than BD-negative ones to be from donors who identified themselves as Hispanic. Among 282 positive donors with country-of-birth data, 33% were from Mexico, 31% from Central America, and 26% from South America. Approximately 10% of donors with country-of-birth data were born in the United States, but the sources of their infections likely represented a mixture (congenital, travel, or locally acquired); this group of donations was not included in the analyses that were based on birth country.

**TABLE 1:** Characteristics of donors whose specimens were used in the evaluation

Characteristic	No. (%) of donors with indicated blood donor status	
	Positive	Negative
Region of blood donation^*a*^		
California	195 (39.0)	115 (38.3)
Other western states	50 (10.0)	31 (10.3)
Southeast	170 (34.0)	103 (34.3)
Midwest	33 (6.6)	19 (6.3)
Northeast	52 (10.4)	32 (10.7)
Sex		
Male	240 (48.0)	127 (42.3)
Female	260 (52.0)	173 (57.7)
Ethnicity		
Hispanic^*b*^	204 (40.8)	37 (12.3)
Caucasian	17 (7.6)	223 (74.3)
Other	1 (0.5)	35 (11.7)
No data	278 (55.6)	5 (1.7)
Region of birth^*c*^		
Mexico	94 (33.3)	
Central America	88 (31.2)	
South America	73 (25.9)	
United States	27 (9.6)	

a Seronegative specimens were frequency matched to seropositive specimens by donation region.

b Blood donors with positive test results were significantly more likely to report Hispanic ethnicity (*P* < 0.0001).

c Data were available for 282 blood donors identified as seropositive in blood donation testing; no data were available for 218 seropositive and 300 seronegative specimens.

The three analyses (BD status, consensus, and LCA) yielded similar results, with overlapping 95% CIs for each parameter across analyses of the same test (Table 2). The highest sensitivity estimates resulted from the LCA and the lowest from the BD comparisons; the reverse trend was seen for specificity. The 2-class LCA showed better fit than a 3-class analysis both by AIC (−2,059.089 versus −2,027.283) and BIC (−2,101.25 versus −2,092.867).

**TABLE 2:** Performance of FDA-cleared Chagas disease IgG serological assays compared to original blood donor status, consensus among current tests, and latent class analysis

Assay^*a*^	Value(s)				
	Blood donor status		Consensus status		Latent class analysis
	Positive	Negative	Positive	Negative	
Hemagen ELISA					
No. of positive specimens	440^*b*^	0	439^*b*^	1	
No. of negative specimens	60	300	45	315	
Sensitivity (%) (95% CI)	88.00 (84.82, 90.72)		90.70 (87.76, 93.14)		92.04 (88.93, 95.04)
Specificity (%) (95% CI)	100.00 (98.88, 100.00)		99.68 (98.25, 99.99)		99.04 (97.20, 100.00)
PPV (%) (95% CI)	100.00 (99.17, 100.00)		99.77 (98.74, 99.99)		
NPV (%) (95% CI)	83.33 (79.07, 87.03)		87.50 (83.63, 90.73)		
Ortho ELISA					
No. of positive specimens	462	0	461	1	
No. of negative specimens	38	300	23	315	
Sensitivity (%) (95% CI)	92.40 (89.72, 94.57)		95.25 (92.95, 96.96)		96.50 (94.20, 99.29)
Specificity (%) (95% CI)	100.00 (98.78, 100.00)		99.68 (98.25, 99.99)		98.82 (96.25, 100.00)
PPV (%) (95% CI)	100.00 (99.20, 100.00)		99.78 (98.80, 99.99)		
NPV (%) (95% CI)	88.76 (84.90, 91.92)		93.20 (89.96, 95.64)		
Wiener ELISA					
No. of positive specimens	470^*c*^	2^*d*^	466^*e*^	6^*f*^	
No. of negative specimens	30	298	18	310	
Sensitivity (%) (95% CI)	94.00 (91.55, 95.92)		96.28 (94.19, 97.78)		97.12 (94.81, 99.24)
Specificity (%) (95% CI)	99.33 (97.61, 99.92)		98.10 (95.91, 99.30)		96.67 (93.92, 98.96)
PPV (%) (95% CI)	99.58 (98.48, 99.95)		98.73 (97.25, 99.53)		
NPV (%) (95% CI)	90.85 (87.20, 93.74)		94.51 (91.47, 96.72)		
InBios CDP					
No. of positive specimens	487	23	480	30	
No. of negative specimens	13	277	4	286	
Sensitivity (%) (95% CI)	97.40 (95.59, 98.61)		99.17 (97.90, 99.77)		99.29 (98.34, 100.00)
Specificity (%) (95% CI)	92.33 (88.82, 95.08)		90.51 (86.72, 93.50)		87.53 (82.24, 91.94)
PPV (%) (95% CI)	95.49 (93.31, 97.12)		94.12 (91.71, 96.00)		
NPV (%) (95% CI)	95.52 (92.46, 97.59)		98.62 (96.51, 99.62)		

a NPV, negative predictive value; PPV, positive predictive value.

b Data include 11 specimens with indeterminate results. Indeterminate results were classified as positive results for the purpose of these analyses (see text for explanation).

c Data include 4 specimens with indeterminate results.

d These 2 specimens had indeterminate results.

e Data include 3 specimens with indeterminate results.

f Data include 3 specimens with indeterminate results.

In all three analyses, InBios CDP had the highest sensitivity (97% to 99%) but the lowest specificity (88% to 92%). Reader agreement on InBios scores was high (weighted kappa = 0.9315; 95% CI, 0.9209 to 0.9420). Agreement on determination of positive (scores 1 to 6) versus negative (score 0) results was higher than 99% (795/800 [99.4%]; kappa = 0.9865; 95% CI, 0.9746 to 0.9983). There were only five discordant results: two specimens positive by reader 1 and negative by reader 2 and three specimens with the converse outcome. The majority of apparent false-positive InBios results had intensity scores of 1 (87% for BD, 83% for consensus analysis). Hemagen displayed the lowest sensitivity (88% to 92%) but high specificity (99% to 100%). Eleven specimens had Hemagen readings in the indeterminate zone; all were BD positive. Sensitivity for the Wiener ELISA ranged from 94% to 97%, with specificity ranging from 97% to 99%. Six specimens, including four BD-positive and two BD-negative specimens, had indeterminate results by Wiener. Of the 500 specimens classified as confirmed positive in BD testing, those with negative results by current assays (apparent false negatives) had significantly lower median Ortho S/CO values in prior BD testing than those with positive results in current testing (apparent true positives) (see Fig. S2 in the supplemental material).

Ortho ELISA sensitivity ranged from 92% to 97% in the current analysis, with specificity of 99% to 100%. Of 500 BD-positive specimens, 489 had positive Ortho results in BD testing; 11 specimens were positive by Abbott PRISM and a supplemental test (RIPA and/or Abbott ESA) but negative by Ortho in BD testing. Four of the 11 previously Ortho-negative specimens had positive results in the current Ortho testing, but 31 previously Ortho-positive specimens had negative results. Current Ortho S/CO values were 15.9% (median) lower than in BD testing (*P* < 0.001). Specimens corresponding to earlier collected donations showed a smaller decline in S/CO values than more recent ones (*Y* = 0.007334 * *X* − 1.534; *R*^2^ = 0.05758; *P* < 0.001 [linear regression analysis of percent decline in S/CO versus specimen age in months]).

**FIG 1:**
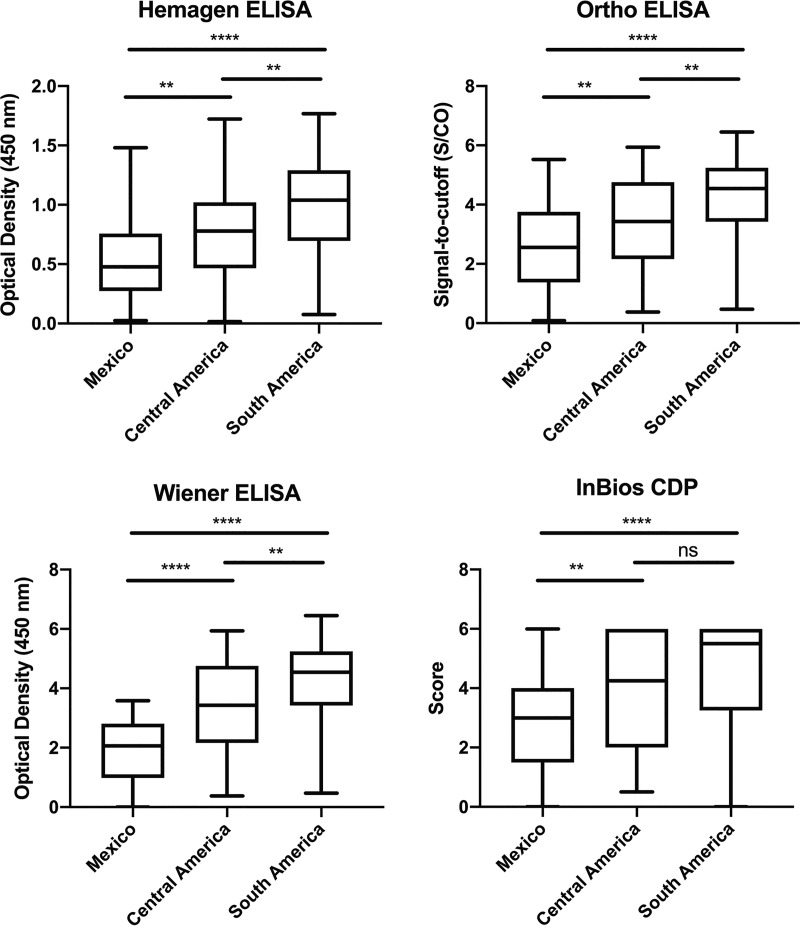
Distribution of positive serology values by blood donor region of birth. Results are expressed as signal over cutoff (S/CO) for Ortho, optical density at 450 nm for Hemagen and Wiener, and scores 0 to 6 for InBios. Across all tests, individuals born in Mexico showed the lowest test values and individuals born in South America the highest. ns, *P* > 0.05; *, *P* ≤ 0.05; **, *P* ≤ 0.01; ***, *P* ≤ 0.001; ****, *P* ≤ 0.0001.

Finally, we stratified results by region of birth to explore geographic variation in test sensitivity (Table 3). Compared to BD or consensus status, sensitivity for Ortho, Wiener, and Hemagen tended to be lowest in specimens from those born in Mexico and highest in those from South America, with Central American specimens showing intermediate results. Analyses of antibody reactivity were consistent with these results, with the lowest reactivity seen in the specimens from Mexico (Fig. 1).

**TABLE 3:** Sensitivity of T. cruzi IgG serological tests by blood donor region of birth

Test	% sensitivity (CI)					
	Blood donor status			Consensus status (at least 2 current tests positive)		
	Mexico	Central America^*a*^	South America^*b*^	Mexico	Central America^*a*^	South America^*b*^
Hemagen	82.98 (74.13, 89.24)	88.64 (80.33, 93.71)	93.15 (84.95, 97.04)	86.67 (78.13, 92.21)	89.66 (89.66, 94.46)	93.15 (84.95, 97.04)
Ortho	85.11 (76.54, 90.92)	95.45 (88.89, 98.22)	97.26 (90.55, 99.51)	88.89 (80.74, 93.82)	96.55 (90.35, 99.06)	97.26 (90.55, 99.51)
Wiener	91.49 (84.10, 95.62)	96.59 (90.45, 99.07)	98.63 (92.64, 99.93)	93.33 (88.84, 91.12)	96.55 (90.35, 99.06)	98.63 (92.64, 99.93)
InBios	97.87 (92.57, 99.62)	98.86 (93.84, 99.94)	98.63 (92.64, 99.93)	100.00 (95.91, 100.00)	100.0 (95.77, 100.00)	98.63 (92.64, 99.93)

a Data represent blood donors born in El Salvador (*n* = 67), Guatemala (*n* = 10), Honduras (*n* = 7), Costa Rica (*n* = 1), Nicaragua (*n* = 1), or an unspecified location in Central America (*n* = 2).

b Data represent donors born in Bolivia (*n* = 32), Argentina (*n* = 13), Chile (*n* = 5), Paraguay (*n* = 2), Uruguay (*n* = 1), Brazil (*n* = 6), Colombia (*n* = 9), Ecuador (*n* = 2), or an unspecified location in South America (*n* = 3).

## DISCUSSION

Our data provide initial evidence for an appropriate diagnostic algorithm for Chagas disease in the United States. The direct comparison of the four FDA-cleared tests demonstrated a range of sensitivity and specificity estimates across tests as well as consistent variation in sensitivity by country of origin. On the basis of these findings, we can develop preliminary guidance for optimal use of these tests, anticipate associated challenges, and identify where improvements are needed.

**FIG 2:**
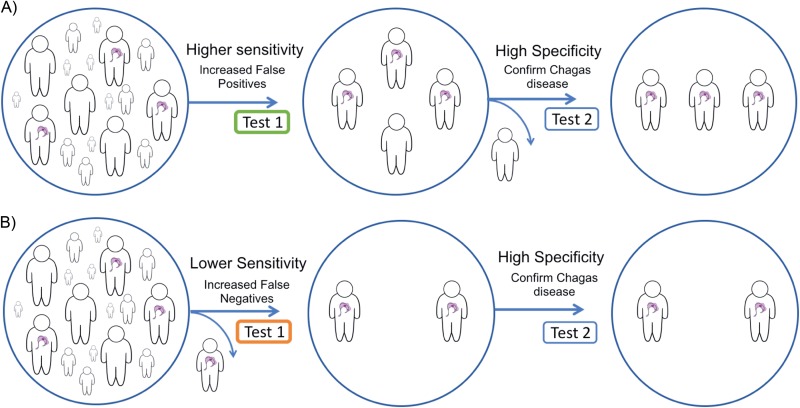
Effects of variations in the clinical sensitivity of initial test in a two-step diagnostic algorithm. Two-step diagnostic algorithms allow an acceptable number of false positives to ensure that positive cases are detected. (A) Higher-sensitivity initial test, with a high-specificity confirmatory test to rule out false positives. (B) Missed case of Chagas disease due to a lower-sensitivity initial test and false-negative result.

In common with recommendations for syphilis and early algorithms for HIV (30, 31), definitive diagnosis of chronic T. cruzi infection requires positive results by two distinct tests (3, 4). This algorithm was developed to address issues of both sensitivity and specificity. Simultaneous use of two tests optimizes both parameters and may be cost-effective in high-prevalence settings. However, when low prevalence is anticipated, universal testing by two assays is impractical. Most programs will use one test as a screen and analyze only the screen positives by the second assay. In these circumstances, the order is crucial; a high-sensitivity screening test is essential to minimize the risk of missing true infections (Fig. 2). At the same time, if specificity is not high, an assay will result in many false positives, potentially undermining confidence in testing. For example, in a setting of 1.5% prevalence (32), any specificity lower than 98.5% will result in more false-positive than true-positive results.

No single test had optimal performance characteristics in our data, despite the high sensitivity and specificity figures reported in their FDA 510(k) clearance applications and package inserts (16, 17, 33, 34). In part, this may be attributable to the differences in performance in a setting closer to “real world” diagnostic testing versus the more controlled setting of a clinical trial. However, a major issue with respect to the available data is that many of the current diagnostic tests were developed using specimen sets from the Southern Cone, where discrete typing units (DTUs) TcII, TcV, and TcVI are most prevalent (17, 33, 34). Only the Ortho evaluations reported results in specimens from at-risk populations in Mexico, Guatemala, and the United States during test development (16, 24). Published data confirm high rates of discordance and false-negative results by other assays in Mexico (21, 23), and the lower antibody reactivity seen in our data poses a challenge to achieving adequate sensitivity. Given the high proportion of U.S. T. cruzi infections with Mexican origins, investigating and addressing the underlying cause of this phenomenon will be central to the effort to improve diagnostic test performance in the United States. TcI, the predominant T. cruzi DTU in Mexico, is widely distributed throughout the Americas (35). TcI also predominates in human infections in northern South America and Central America (36). Thus, the low reactivity in Central America compared to South America may be linked to differences between TcI and TcII, TcV, and TcVI, but the markedly lower reactivity in Mexican specimens was not solely a result of TcI predominance. Poorly understood strain differences within the TcI DTU may also be responsible for the observed geographic variability in immune response (20, 22).

On the basis of the performances reflected in our data, the Wiener Recombinante 3.0 and Ortho ELISAs showed the best balance of sensitivity and specificity, but both had suboptimal sensitivity in Mexican specimens. The InBios rapid test had the best sensitivity, with high sensitivity even in Mexican specimens, but its low specificity would result in a substantial number of false positives requiring confirmatory testing. The low sensitivity of Hemagen, especially in Mexican specimens, raises the risk of false negatives and concerns for its use as a screening test. In all cases, discordant results between screening and confirmatory testing should prompt the use of a third test as a tie-breaker, such as the IgG trypomastigote excreted-secreted antigen (TESA) blot or the Abbott ESA, the latter having received FDA licensure for confirmatory use in the blood donor screening algorithm.

The use of surplus blood donation specimens has both limitations and advantages. Blood donor populations are not representative of the general U.S. population; donors are younger and healthier than the population at large, and although the rate of donation by Hispanics has increased markedly over the past decade, this group remains underrepresented (37, 38). However, given the design of the study, these differences should not affect the validity of the test performance estimates. Although three of the four tests are validated for both serum and plasma, the Hemagen package insert specifies the use of serum; we had only plasma available, which may have had an impact on our estimates for this assay. However, the other T. cruzi serology kits and other similar assays for infectious diseases have reported equivalent results between serum and plasma (39, 40). The decrease in reactivity by the Ortho ELISA in current versus prior BD testing is perplexing. Length of storage was inversely related to the magnitude of the decline, making antibody degradation an unlikely explanation. The Ortho ELISA uses cultured parasite lysate as its antigen source, possibly introducing biological variability.

A critical review of diagnostic studies suggests that a double-blinded prospective cohort provides the optimal study design, because testing of positive and negative groups selected on the basis of prior test results introduces a bias toward overestimates of performance characteristics, especially if discordant specimens are excluded (41). However, prospective testing by multiple assays in a very-low-prevalence population would incur prohibitive costs. Ortho ELISA was the BD screening test for many of the specimens and was also part of the evaluation; this was unavoidable, given that the assay is approved for both applications, but raises the potential for selection bias. We attempted to minimize bias by incorporating specimens that were discordant in BD testing and specimens across the entire range of antibody responses and by using two different comparators (BD and consensus) and a latent class analysis. Our results demonstrate how performance estimates may vary depending on the comparator and analysis method. Our study design was strengthened by the large sample size, which included specimens across the spectrum of reactivity levels and infections acquired in different geographic regions, characteristics difficult to replicate in the United States in the absence of a large, well-funded multicenter study. Our results do not preclude such a study. On the contrary, additional rigorous analyses of data from robust specimen sets with broad geographic coverage are essential to better understand and improve the performance of the available tests in U.S. populations at risk of T. cruzi infection.

### Conclusion.

In an analysis of U.S. blood donor specimens, the InBios Chagas Detect Plus rapid test had the highest sensitivity but lowest specificity, while the Hemagen assay had the lowest sensitivity among the FDA-cleared tests. The Hemagen, Ortho, and Wiener ELISAs all had equivalently high levels of specificity. Sensitivity was lowest for the Ortho, Wiener, and Hemagen ELISAs in specimens from donors born in Mexico, intermediate for those born in Central America, and highest for those born in South America, consistent with differences in the distributions of antibody reactivity in these groups. Use of a high-sensitivity screening test, followed by a second higher-specificity test, offers the best current algorithm for diagnostic screening in the United States.

## Supplementary Material

Supplemental file 1
