# Supplementary material for: Chagas Disease Serological Test Performance in U.S. Blood Donor Specimens
Source: J Clin Microbiol. 2019 Nov 22;57(12):e01217-19. doi: 10.1128/JCM.01217-19 (PMC6879282; doi:10.1128/JCM.01217-19)
Supplement: Supplemental file 1 [file JCM.01217-19-s0001.pdf]

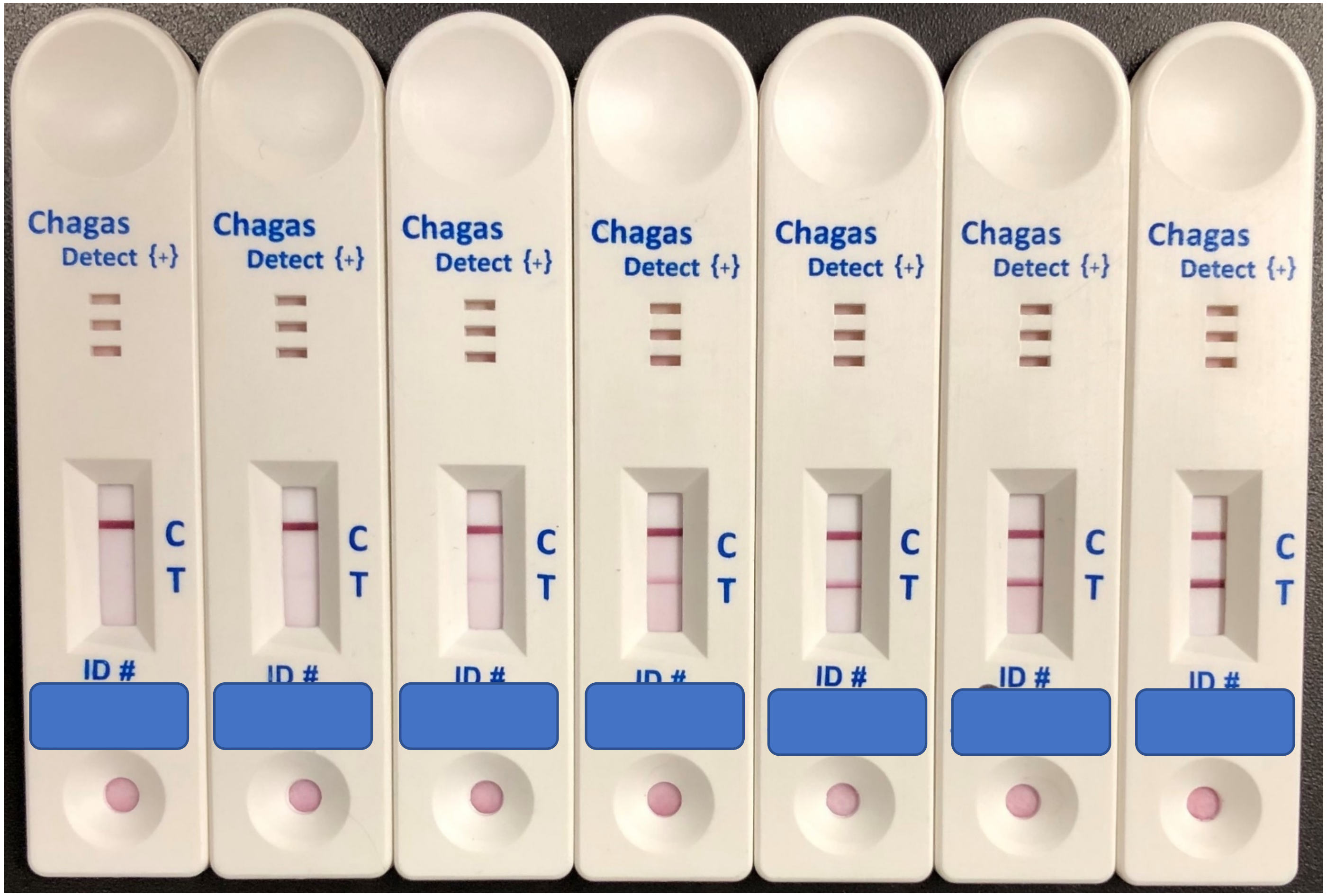

0 1 2 3 4 5 6

Figure S1. Scoring template for InBios ChagasDetect Plus readings

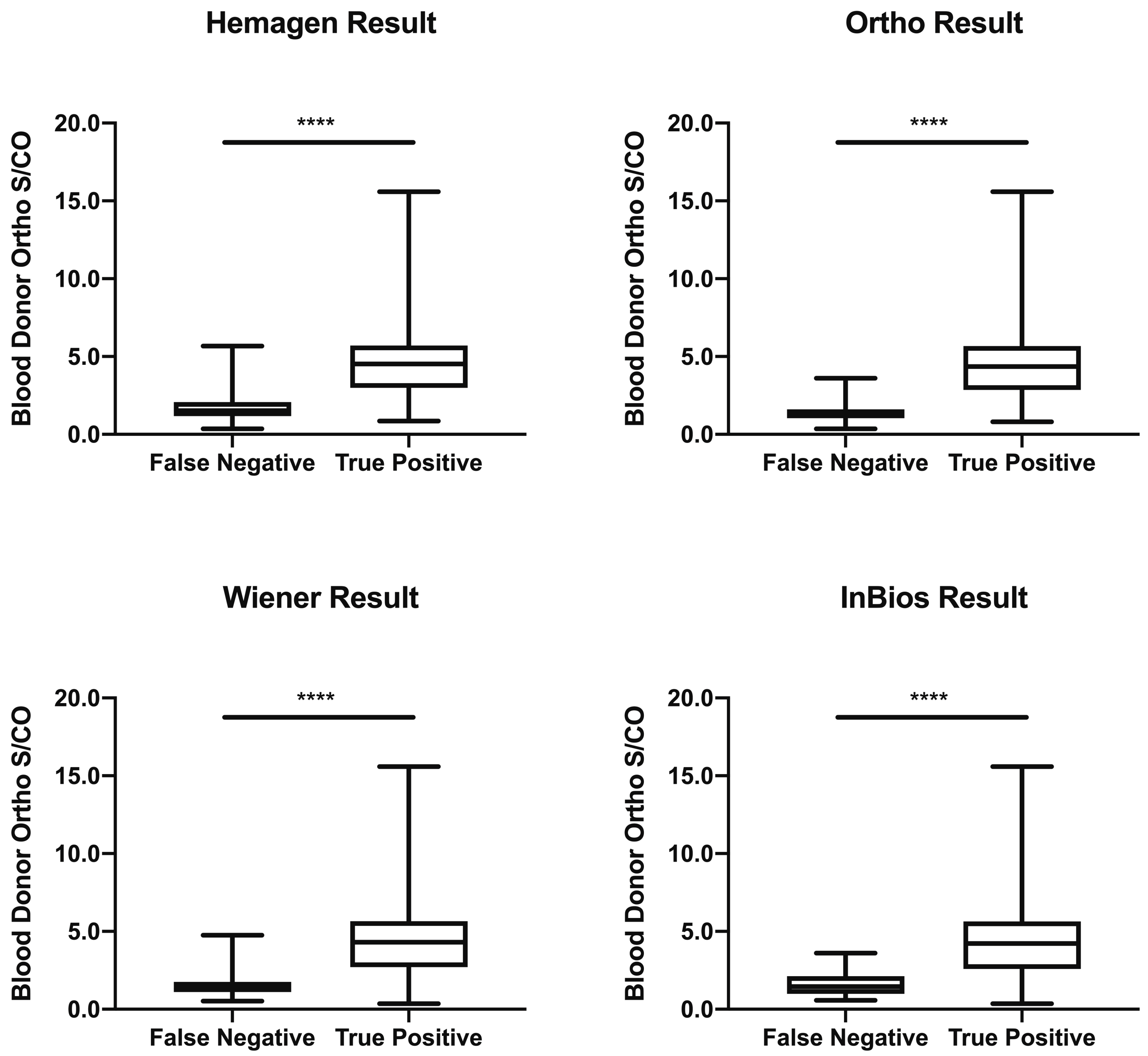

Figure S2. Blood donor Ortho signal-to-cutoff (S/CO) distribution for specimens with false negative and true positive designation by test as compared to blood donor status. ****p<0.0001
